# Supplementary material for: Pulmonary MicroRNA Changes Alter Angiogenesis in Chronic Obstructive Pulmonary Disease and Lung Cancer
Source: Biomedicines. 2021 Jul 16;9(7):830. doi: 10.3390/biomedicines9070830 (PMC8301412; doi:10.3390/biomedicines9070830)
Supplement: Supplementary file 1 [file biomedicines-09-00830-s001.zip › biomedicines-1272798-supplementary.pdf]

Pulmonary microRNA changes alter angiogenesis in chronic obstructive pulmonary disease and lung cancer

CE Green, J Clarke, R Bicknell & AM Turner

Online Data Supplement

A

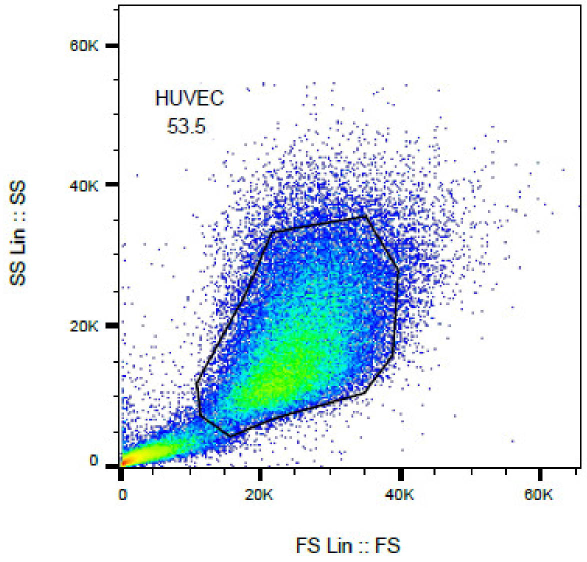

B

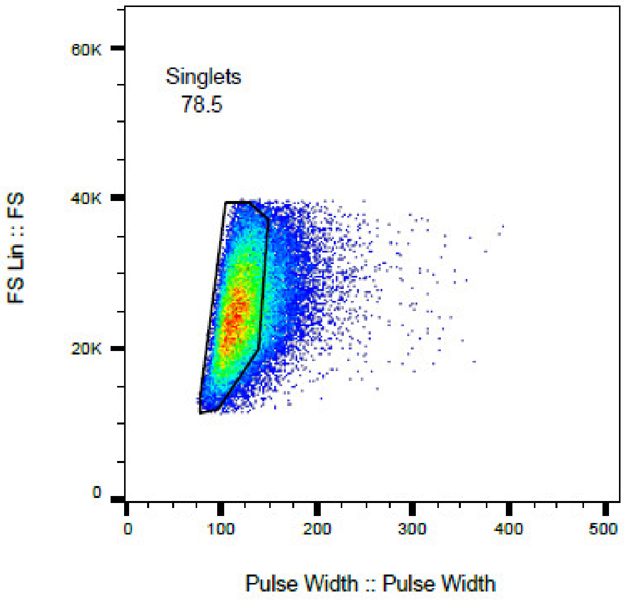

Figure S1. Cytogram plots from the program 'FloJo' used for cell cycle analysis. (**A**): An initial gate was drawn to identify the main cell population. (**B**): A second gate was drawn to identify single cells within the main cell population. The single cell population was used for cell cycle analysis.

RESULTS

Table S1. Significantly upregulated miR in COPD.

Gene ID			
hsa-let-7f-1-3p	hsa-miR-1264	hsa-miR-146a-3p	hsa-miR-1914-5p
hsa-let-7f-2-3p	hsa-miR-1267	hsa-miR-146a-5p	hsa-miR-193b-3p
hsa-let-7i-3p	hsa-miR-1269a	hsa-miR-146b-3p	hsa-miR-193b-5p
hsa-miR-1	hsa-miR-1269b	hsa-miR-146b-5p	hsa-miR-196a-3p
hsa-miR-100-5p	hsa-miR-127-3p	hsa-miR-1470	hsa-miR-196b-5p
hsa-miR-103a-2-5p	hsa-miR-1273a	hsa-miR-148a-3p	hsa-miR-199a-5p
hsa-miR-103b	hsa-miR-1273d	hsa-miR-148a-5p	hsa-miR-199b-5p
hsa-miR-105-5p	hsa-miR-1273g-5p	hsa-miR-148b-3p	hsa-miR-19a-3p
hsa-miR-10a-3p	hsa-miR-1277-3p	hsa-miR-148b-5p	hsa-miR-19b-3p
hsa-miR-10b-5p	hsa-miR-1281	hsa-miR-149-5p	hsa-miR-200a-3p
hsa-miR-1178-5p	hsa-miR-1282	hsa-miR-150-3p	hsa-miR-200a-5p
hsa-miR-1184	hsa-miR-1283	hsa-miR-152	hsa-miR-200b-3p
hsa-miR-1185-5p	hsa-miR-1288	hsa-miR-153	hsa-miR-200c-3p
hsa-miR-1200	hsa-miR-1289	hsa-miR-1538	hsa-miR-200c-5p
hsa-miR-1204	hsa-miR-129-2-3p	hsa-miR-154-3p	hsa-miR-202-3p
hsa-miR-1206	hsa-miR-1292-3p	hsa-miR-154-5p	hsa-miR-202-5p
hsa-miR-1225-5p	hsa-miR-1293	hsa-miR-155-3p	hsa-miR-203a
hsa-miR-1226-5p	hsa-miR-1294	hsa-miR-155-5p	hsa-miR-203b-3p
hsa-miR-1228-5p	hsa-miR-1295a	hsa-miR-16-2-3p	hsa-miR-204-5p
hsa-miR-1229-3p	hsa-miR-1295b-5p	hsa-miR-17-3p	hsa-miR-206
hsa-miR-1231	hsa-miR-1296	hsa-miR-181a-5p	hsa-miR-208a
hsa-miR-1234-3p	hsa-miR-1298	hsa-miR-181b-3p	hsa-miR-208b
hsa-miR-1234-5p	hsa-miR-1303	hsa-miR-181c-5p	hsa-miR-20b-3p
hsa-miR-1238-3p	hsa-miR-1304-5p	hsa-miR-182-3p	hsa-miR-21-3p
hsa-miR-1238-5p	hsa-miR-1306-5p	hsa-miR-183-5p	hsa-miR-21-5p
hsa-miR-1245b-3p	hsa-miR-1307-3p	hsa-miR-184	hsa-miR-2110
hsa-miR-1247-5p	hsa-miR-1307-5p	hsa-miR-185-3p	hsa-miR-2114-5p
hsa-miR-1248	hsa-miR-130a-5p	hsa-miR-186-3p	hsa-miR-2115-3p
hsa-miR-1250	hsa-miR-130b-3p	hsa-miR-187-3p	hsa-miR-2115-5p
hsa-miR-1251	hsa-miR-132-3p	hsa-miR-188-3p	hsa-miR-2116-3p
hsa-miR-1252	hsa-miR-135a-5p	hsa-miR-18a-3p	hsa-miR-2117
hsa-miR-1253	hsa-miR-135b-5p	hsa-miR-18a-5p	hsa-miR-212-5p
hsa-miR-1255b-5p	hsa-miR-136-3p	hsa-miR-18b-3p	hsa-miR-216a-3p
hsa-miR-1256	hsa-miR-136-5p	hsa-miR-18b-5p	hsa-miR-216a-5p
hsa-miR-1257	hsa-miR-138-5p	hsa-miR-1909-3p	hsa-miR-218-2-3p
hsa-miR-125a-5p	hsa-miR-141-3p	hsa-miR-190a	hsa-miR-219-1-3p
hsa-miR-125b-2-3p	hsa-miR-141-5p	hsa-miR-1910	hsa-miR-219-2-3p
hsa-miR-1260a	hsa-miR-142-3p	hsa-miR-1911-3p	hsa-miR-22-5p
hsa-miR-1260b	hsa-miR-142-5p	hsa-miR-1912	hsa-miR-221-3p
hsa-miR-1262	hsa-miR-144-5p	hsa-miR-1913	hsa-miR-221-5p
hsa-miR-222-5p	hsa-miR-3140-5p	hsa-miR-33a-5p	hsa-miR-3681-3p
hsa-miR-223-5p	hsa-miR-3142	hsa-miR-33b-3p	hsa-miR-3681-5p
hsa-miR-224-3p	hsa-miR-3144-3p	hsa-miR-33b-5p	hsa-miR-3683
hsa-miR-224-5p	hsa-miR-3145-3p	hsa-miR-340-3p	hsa-miR-3685
hsa-miR-2277-5p	hsa-miR-3145-5p	hsa-miR-340-5p	hsa-miR-3687

hsa-miR-2355-3p	hsa-miR-3146	hsa-miR-342-5p	hsa-miR-3688-5p
hsa-miR-23c	hsa-miR-3152-3p	hsa-miR-34a-3p	hsa-miR-3689a-3p
hsa-miR-2467-5p	hsa-miR-3152-5p	hsa-miR-34a-5p	hsa-miR-3689a-5p
hsa-miR-2681-3p	hsa-miR-3155a	hsa-miR-34b-5p	hsa-miR-3689d
hsa-miR-2681-5p	hsa-miR-3156-3p	hsa-miR-34c-3p	hsa-miR-3689f
hsa-miR-2682-5p	hsa-miR-3156-5p	hsa-miR-3529-3p	hsa-miR-369-3p
hsa-miR-26a-1-3p	hsa-miR-3157-3p	hsa-miR-3591-3p	hsa-miR-369-5p
hsa-miR-26b-3p	hsa-miR-3158-5p	hsa-miR-3607-3p	hsa-miR-3690
hsa-miR-27a-5p	hsa-miR-3160-5p	hsa-miR-3607-5p	hsa-miR-3691-5p
hsa-miR-296-3p	hsa-miR-3161	hsa-miR-3611	hsa-miR-3714
hsa-miR-296-5p	hsa-miR-3166	hsa-miR-3612	hsa-miR-371a-3p
hsa-miR-2964a-3p	hsa-miR-3167	hsa-miR-3613-5p	hsa-miR-371b-3p
hsa-miR-29b-2-5p	hsa-miR-3173-5p	hsa-miR-3619-3p	hsa-miR-374a-3p
hsa-miR-29b-3p	hsa-miR-3175	hsa-miR-3619-5p	hsa-miR-374a-5p
hsa-miR-301a-5p	hsa-miR-3176	hsa-miR-362-3p	hsa-miR-374b-3p
hsa-miR-301b	hsa-miR-3179	hsa-miR-362-5p	hsa-miR-374c-3p
hsa-miR-302a-3p	hsa-miR-3180	hsa-miR-3620-3p	hsa-miR-374c-5p
hsa-miR-302a-5p	hsa-miR-3181	hsa-miR-3622a-3p	hsa-miR-375
hsa-miR-302b-3p	hsa-miR-3182	hsa-miR-363-5p	hsa-miR-376a-2-5p
hsa-miR-302c-3p	hsa-miR-3183	hsa-miR-3649	hsa-miR-376a-3p
hsa-miR-302d-3p	hsa-miR-3184-5p	hsa-miR-3650	hsa-miR-376a-5p
hsa-miR-302e	hsa-miR-3185	hsa-miR-3651	hsa-miR-376b-3p
hsa-miR-302f	hsa-miR-3191-3p	hsa-miR-3652	hsa-miR-376c-3p
hsa-miR-3064-3p	hsa-miR-3192	hsa-miR-3653	hsa-miR-376c-5p
hsa-miR-3065-5p	hsa-miR-3193	hsa-miR-3654	hsa-miR-377-3p
hsa-miR-3115	hsa-miR-3197	hsa-miR-3655	hsa-miR-377-5p
hsa-miR-3116	hsa-miR-32-3p	hsa-miR-3658	hsa-miR-378a-3p
hsa-miR-3118	hsa-miR-3200-3p	hsa-miR-3660	hsa-miR-378a-5p
hsa-miR-3119	hsa-miR-3200-5p	hsa-miR-3662	hsa-miR-378d
hsa-miR-3120-5p	hsa-miR-3201	hsa-miR-3664-3p	hsa-miR-378g
hsa-miR-3121-5p	hsa-miR-320d	hsa-miR-3669	hsa-miR-378h
hsa-miR-3123	hsa-miR-323a-3p	hsa-miR-3670	hsa-miR-378i
hsa-miR-3124-3p	hsa-miR-323a-5p	hsa-miR-3671	hsa-miR-378j
hsa-miR-3124-5p	hsa-miR-323b-3p	hsa-miR-3674	hsa-miR-379-3p
hsa-miR-3126-3p	hsa-miR-323b-5p	hsa-miR-3675-3p	hsa-miR-379-5p
hsa-miR-3127-3p	hsa-miR-324-5p	hsa-miR-3677-3p	hsa-miR-381-3p
hsa-miR-3129-5p	hsa-miR-330-5p	hsa-miR-3677-5p	hsa-miR-381-5p
hsa-miR-3130-5p	hsa-miR-331-5p	hsa-miR-3678-5p	hsa-miR-382-3p
hsa-miR-3133	hsa-miR-335-5p	hsa-miR-3680-3p	hsa-miR-3908
hsa-miR-3134	hsa-miR-339-5p	hsa-miR-3680-5p	hsa-miR-3910
hsa-miR-3912	hsa-miR-4301	hsa-miR-448	hsa-miR-454-3p
hsa-miR-3913-3p	hsa-miR-4307	hsa-miR-4483	hsa-miR-454-5p
hsa-miR-3914	hsa-miR-4308	hsa-miR-4486	hsa-miR-4540
hsa-miR-3915	hsa-miR-4312	hsa-miR-4488	hsa-miR-4632-3p
hsa-miR-3916	hsa-miR-4315	hsa-miR-4489	hsa-miR-4633-5p
hsa-miR-3920	hsa-miR-4318	hsa-miR-4491	hsa-miR-4636
hsa-miR-3921	hsa-miR-4319	hsa-miR-4493	hsa-miR-4637
hsa-miR-3922-3p	hsa-miR-432-3p	hsa-miR-4494	hsa-miR-4639-3p
hsa-miR-3922-5p	hsa-miR-4320	hsa-miR-4498	hsa-miR-4639-5p
hsa-miR-3924	hsa-miR-4321	hsa-miR-449a	hsa-miR-4641
hsa-miR-3925-3p	hsa-miR-4322	hsa-miR-449b-3p	hsa-miR-4642

hsa-miR-3927-5p	hsa-miR-4323	hsa-miR-449b-5p	hsa-miR-4643
hsa-miR-3929	hsa-miR-4325	hsa-miR-449c-5p	hsa-miR-4649-5p
hsa-miR-3936	hsa-miR-4326	hsa-miR-4501	hsa-miR-4650-5p
hsa-miR-3941	hsa-miR-4329	hsa-miR-4503	hsa-miR-4652-5p
hsa-miR-3944-5p	hsa-miR-4330	hsa-miR-4504	hsa-miR-4653-5p
hsa-miR-3972	hsa-miR-4418	hsa-miR-4506	hsa-miR-4656
hsa-miR-3973	hsa-miR-4419b	hsa-miR-4508	hsa-miR-4657
hsa-miR-3974	hsa-miR-4423-5p	hsa-miR-4509	hsa-miR-466
hsa-miR-3975	hsa-miR-4424	hsa-miR-450a-3p	hsa-miR-4661-3p
hsa-miR-3978	hsa-miR-4425	hsa-miR-450a-5p	hsa-miR-4662a-3p
hsa-miR-409-3p	hsa-miR-4426	hsa-miR-450b-3p	hsa-miR-4662a-5p
hsa-miR-411-3p	hsa-miR-4427	hsa-miR-450b-5p	hsa-miR-4662b
hsa-miR-411-5p	hsa-miR-4429	hsa-miR-4510	hsa-miR-4666a-3p
hsa-miR-423-3p	hsa-miR-4432	hsa-miR-4511	hsa-miR-4667-3p
hsa-miR-4254	hsa-miR-4434	hsa-miR-4512	hsa-miR-4668-3p
hsa-miR-4258	hsa-miR-4436b-3p	hsa-miR-4517	hsa-miR-4671-3p
hsa-miR-4262	hsa-miR-4438	hsa-miR-4519	hsa-miR-4671-5p
hsa-miR-4264	hsa-miR-4441	hsa-miR-451b	hsa-miR-4673
hsa-miR-4270	hsa-miR-4445-3p	hsa-miR-452-5p	hsa-miR-4674
hsa-miR-4272	hsa-miR-4445-5p	hsa-miR-4520a-3p	hsa-miR-4675
hsa-miR-4273	hsa-miR-4446-5p	hsa-miR-4520b-3p	hsa-miR-4680-3p
hsa-miR-4275	hsa-miR-4447	hsa-miR-4521	hsa-miR-4680-5p
hsa-miR-4276	hsa-miR-4449	hsa-miR-4523	hsa-miR-4682
hsa-miR-4277	hsa-miR-4450	hsa-miR-4524a-3p	hsa-miR-4683
hsa-miR-4278	hsa-miR-4454	hsa-miR-4524b-3p	hsa-miR-4684-3p
hsa-miR-4281	hsa-miR-4457	hsa-miR-4525	hsa-miR-4686
hsa-miR-4282	hsa-miR-4460	hsa-miR-4526	hsa-miR-4687-5p
hsa-miR-4285	hsa-miR-4467	hsa-miR-4527	hsa-miR-4690-3p
hsa-miR-4287	hsa-miR-4473	hsa-miR-4528	hsa-miR-4693-3p
hsa-miR-4288	hsa-miR-4474-3p	hsa-miR-4532	hsa-miR-4694-5p
hsa-miR-429	hsa-miR-4474-5p	hsa-miR-4536-5p	hsa-miR-4695-3p
hsa-miR-4291	hsa-miR-4477a	hsa-miR-4537	hsa-miR-4697-3p
hsa-miR-4292	hsa-miR-4477b	hsa-miR-4538	hsa-miR-4700-5p
hsa-miR-4293	hsa-miR-4479	hsa-miR-4539	hsa-miR-4701-3p
hsa-miR-4703-5p	hsa-miR-4761-3p	hsa-miR-488-5p	hsa-miR-516a-5p
hsa-miR-4704-3p	hsa-miR-4761-5p	hsa-miR-489	hsa-miR-517a-3p
hsa-miR-4704-5p	hsa-miR-4762-3p	hsa-miR-490-5p	hsa-miR-517c-3p
hsa-miR-4707-3p	hsa-miR-4762-5p	hsa-miR-491-3p	hsa-miR-5187-5p
hsa-miR-4708-3p	hsa-miR-4763-3p	hsa-miR-493-5p	hsa-miR-5189
hsa-miR-4709-5p	hsa-miR-4763-5p	hsa-miR-495-3p	hsa-miR-518b
hsa-miR-4711-5p	hsa-miR-4764-3p	hsa-miR-497-3p	hsa-miR-518c-3p
hsa-miR-4712-5p	hsa-miR-4765	hsa-miR-4999-5p	hsa-miR-518c-5p
hsa-miR-4713-5p	hsa-miR-4766-3p	hsa-miR-499a-5p	hsa-miR-518d-3p
hsa-miR-4714-3p	hsa-miR-4766-5p	hsa-miR-499b-3p	hsa-miR-5191
hsa-miR-4714-5p	hsa-miR-4768-5p	hsa-miR-5000-3p	hsa-miR-5193
hsa-miR-4715-5p	hsa-miR-4770	hsa-miR-5000-5p	hsa-miR-5195-5p
hsa-miR-4717-5p	hsa-miR-4771	hsa-miR-5001-3p	hsa-miR-5196-3p
hsa-miR-4718	hsa-miR-4772-3p	hsa-miR-5002-5p	hsa-miR-5197-3p
hsa-miR-4719	hsa-miR-4772-5p	hsa-miR-5004-3p	hsa-miR-519b-3p
hsa-miR-4720-3p	hsa-miR-4776-5p	hsa-miR-5004-5p	hsa-miR-519c-3p
hsa-miR-4722-3p	hsa-miR-4777-3p	hsa-miR-5007-3p	hsa-miR-519d

hsa-miR-4723-3p	hsa-miR-4777-5p	hsa-miR-5007-5p	hsa-miR-519e-3p
hsa-miR-4725-3p	hsa-miR-4778-3p	hsa-miR-5008-3p	hsa-miR-520a-3p
hsa-miR-4726-3p	hsa-miR-4779	hsa-miR-5009-3p	hsa-miR-520a-5p
hsa-miR-4727-3p	hsa-miR-4781-3p	hsa-miR-500a-3p	hsa-miR-520b
hsa-miR-4727-5p	hsa-miR-4781-5p	hsa-miR-5010-5p	hsa-miR-520c-3p
hsa-miR-4728-3p	hsa-miR-4782-3p	hsa-miR-5011-3p	hsa-miR-520f
hsa-miR-4728-5p	hsa-miR-4782-5p	hsa-miR-5011-5p	hsa-miR-520h
hsa-miR-4729	hsa-miR-4783-3p	hsa-miR-502-3p	hsa-miR-526b-3p
hsa-miR-4731-5p	hsa-miR-4783-5p	hsa-miR-503-5p	hsa-miR-532-3p
hsa-miR-4732-3p	hsa-miR-4789-3p	hsa-miR-505-5p	hsa-miR-532-5p
hsa-miR-4733-3p	hsa-miR-4789-5p	hsa-miR-506-3p	hsa-miR-539-3p
hsa-miR-4733-5p	hsa-miR-4790-5p	hsa-miR-507	hsa-miR-539-5p
hsa-miR-4735-3p	hsa-miR-4795-3p	hsa-miR-5089-3p	hsa-miR-542-3p
hsa-miR-4738-5p	hsa-miR-4795-5p	hsa-miR-5089-5p	hsa-miR-543
hsa-miR-4740-3p	hsa-miR-4797-3p	hsa-miR-509-5p	hsa-miR-544b
hsa-miR-4740-5p	hsa-miR-4797-5p	hsa-miR-5090	hsa-miR-545-5p
hsa-miR-4742-5p	hsa-miR-4798-3p	hsa-miR-5091	hsa-miR-548aa
hsa-miR-4743-3p	hsa-miR-4798-5p	hsa-miR-5094	hsa-miR-548ac
hsa-miR-4744	hsa-miR-4799-3p	hsa-miR-5095	hsa-miR-548ad
hsa-miR-4745-3p	hsa-miR-4799-5p	hsa-miR-511	hsa-miR-548ae
hsa-miR-4752	hsa-miR-4802-3p	hsa-miR-512-3p	hsa-miR-548ah-5p
hsa-miR-4753-3p	hsa-miR-4803	hsa-miR-512-5p	hsa-miR-548ai
hsa-miR-4755-3p	hsa-miR-4804-5p	hsa-miR-513a-3p	hsa-miR-548aj-5p
hsa-miR-4755-5p	hsa-miR-483-3p	hsa-miR-513c-5p	hsa-miR-548ak
hsa-miR-4756-3p	hsa-miR-485-3p	hsa-miR-514a-5p	hsa-miR-548am-5p
hsa-miR-4757-3p	hsa-miR-486-3p	hsa-miR-514b-3p	hsa-miR-548ao-3p
hsa-miR-4757-5p	hsa-miR-487b	hsa-miR-514b-5p	hsa-miR-548ao-5p
hsa-miR-4760-5p	hsa-miR-488-3p	hsa-miR-515-5p	hsa-miR-548ap-3p
hsa-miR-548aq-5p	hsa-miR-5686	hsa-miR-6128	hsa-miR-6514-5p
hsa-miR-548as-3p	hsa-miR-5688	hsa-miR-613	hsa-miR-653
hsa-miR-548at-3p	hsa-miR-5692b	hsa-miR-6133	hsa-miR-654-3p
hsa-miR-548au-3p	hsa-miR-5692c	hsa-miR-6134	hsa-miR-655
hsa-miR-548au-5p	hsa-miR-5693	hsa-miR-614	hsa-miR-656
hsa-miR-548ax	hsa-miR-5694	hsa-miR-616-5p	hsa-miR-657
hsa-miR-548ay-5p	hsa-miR-5695	hsa-miR-618	hsa-miR-658
hsa-miR-548az-3p	hsa-miR-5697	hsa-miR-620	hsa-miR-659-5p
hsa-miR-548az-5p	hsa-miR-5698	hsa-miR-624-3p	hsa-miR-660-3p
hsa-miR-548b-3p	hsa-miR-570-3p	hsa-miR-624-5p	hsa-miR-661
hsa-miR-548b-5p	hsa-miR-5700	hsa-miR-626	hsa-miR-662
hsa-miR-548d-5p	hsa-miR-5704	hsa-miR-627	hsa-miR-664a-3p
hsa-miR-548e	hsa-miR-5705	hsa-miR-628-3p	hsa-miR-664b-3p
hsa-miR-548f	hsa-miR-5706	hsa-miR-628-5p	hsa-miR-668
hsa-miR-548g-3p	hsa-miR-5707	hsa-miR-632	hsa-miR-671-3p
hsa-miR-548j	hsa-miR-571	hsa-miR-633	hsa-miR-6715a-3p
hsa-miR-548k	hsa-miR-573	hsa-miR-634	hsa-miR-6715b-3p
hsa-miR-548n	hsa-miR-576-3p	hsa-miR-635	hsa-miR-6716-3p
hsa-miR-548p	hsa-miR-578	hsa-miR-636	hsa-miR-6717-5p
hsa-miR-548t-5p	hsa-miR-580	hsa-miR-637	hsa-miR-6721-5p
hsa-miR-548u	hsa-miR-582-3p	hsa-miR-641	hsa-miR-6722-5p
hsa-miR-549a	hsa-miR-582-5p	hsa-miR-642a-5p	hsa-miR-675-3p
hsa-miR-550b-2-5p	hsa-miR-584-3p	hsa-miR-642b-5p	hsa-miR-675-5p

hsa-miR-550b-3p	hsa-miR-585	hsa-miR-643	hsa-miR-676-3p
hsa-miR-553	hsa-miR-588	hsa-miR-644a	hsa-miR-7-1-3p
hsa-miR-554	hsa-miR-589-3p	hsa-miR-645	hsa-miR-7-2-3p
hsa-miR-556-3p	hsa-miR-589-5p	hsa-miR-647	hsa-miR-708-5p
hsa-miR-5579-5p	hsa-miR-590-5p	hsa-miR-649	hsa-miR-758-3p
hsa-miR-5580-3p	hsa-miR-591	hsa-miR-6499-5p	hsa-miR-759
hsa-miR-5582-3p	hsa-miR-593-3p	hsa-miR-650	hsa-miR-761
hsa-miR-5583-5p	hsa-miR-595	hsa-miR-6500-5p	hsa-miR-764
hsa-miR-5589-3p	hsa-miR-597	hsa-miR-6501-5p	hsa-miR-766-5p
hsa-miR-5589-5p	hsa-miR-599	hsa-miR-6502-5p	hsa-miR-769-5p
hsa-miR-5590-5p	hsa-miR-603	hsa-miR-6503-3p	hsa-miR-877-3p
hsa-miR-5591-5p	hsa-miR-605	hsa-miR-6503-5p	hsa-miR-877-5p
hsa-miR-561-3p	hsa-miR-606	hsa-miR-6504-3p	hsa-miR-885-3p
hsa-miR-561-5p	hsa-miR-6075	hsa-miR-6504-5p	hsa-miR-888-5p
hsa-miR-563	hsa-miR-6078	hsa-miR-6505-5p	hsa-miR-890
hsa-miR-566	hsa-miR-6082	hsa-miR-6506-3p	hsa-miR-891a
hsa-miR-567	hsa-miR-6084	hsa-miR-6506-5p	hsa-miR-892c-5p
hsa-miR-568	hsa-miR-6085	hsa-miR-6507-3p	hsa-miR-9-3p
hsa-miR-5680	hsa-miR-6086	hsa-miR-6509-5p	hsa-miR-921
hsa-miR-5681a	hsa-miR-609	hsa-miR-6511a-3p	hsa-miR-92a-1-5p
hsa-miR-5681b	hsa-miR-611	hsa-miR-6512-3p	hsa-miR-92b-5p
hsa-miR-5684	hsa-miR-612	hsa-miR-6513-5p	hsa-miR-937-3p
hsa-miR-938	hsa-miR-941	hsa-miR-943	hsa-miR-944

This table demonstrates the miR that were significantly upregulated in COPD vs non-COPD in the miR microarray analyses.

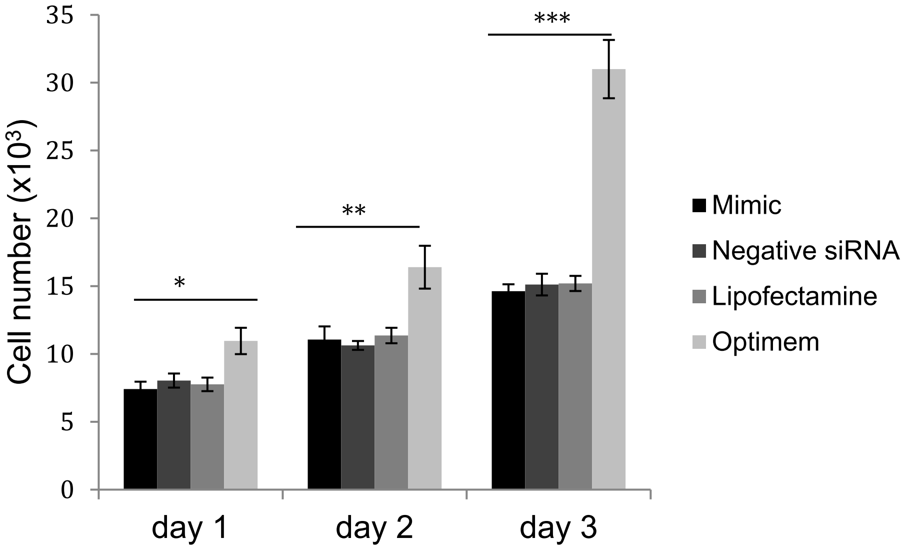

Figure S2. Cell growth assay. This figure shows the number of cells day 1, 2 and 3 after transfection in cells transfected with miR mimic, negative siRNA, lipofectamine or optimem. Numbers represent the mean from three separate experiments. There was no significant difference between mimic and negative siRNA groups suggesting that the miR mimic did not affect cell growth. The Kruskal-Wallis test was used to determine significant differences between groups. $n = 3$ in each group. Day 1; $p = 0.046$, day 2; $p = 0.001$, day 3; op < 0.001. * = p <0.05, ** = p <0.01, *** = p <0.001.

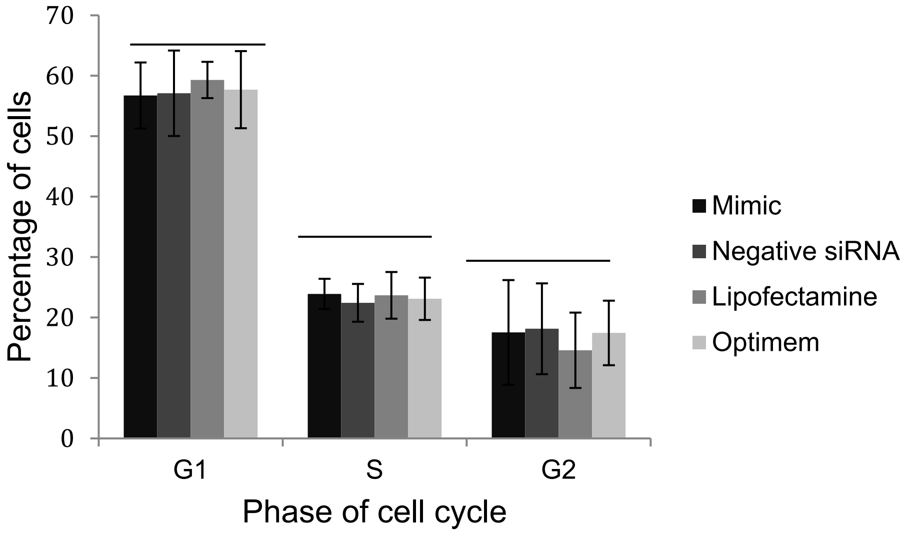

Figure S3. Cell cycle analysis according to flow cytometry. The figure shows the proportion of cells in G1, S and G2 phases of the cell cycle in cells transfected with miR mimic, negative siRNA, lipofectamine or optimem. The values represent the mean from three separate experiments. There was no significant difference between mimic and negative siRNA groups suggesting that the miR mimic did not affect the cell cycle. A one-way ANOVA test was used to determine significant differences between groups. Tukey's test was used post-hoc to look for significant differences between individual groups. There were no significant differences detected. $n = 3$ in each group.

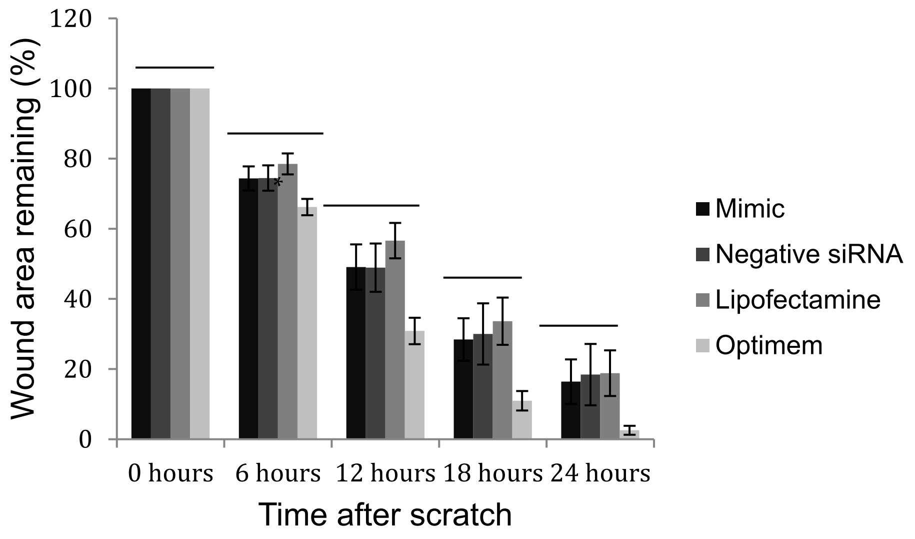

Figure S4. Percentage wound area remaining during scratch wound assay using a miR-181b-3p mimic. The average percentage wound remaining for each condition is recorded at 0, 6, 12, 18 and 24 h. The data represents the mean from three separate experiments. An ANOVA test was used to look for significance between groups. $p = 0.021$ at 12 h. * = $p < 0.05$ (ANOVA). A Tukey's test demonstrated that there was no significant difference between the mimic and negative siRNA groups suggesting that miR-181b-3p does not affect wound adhesion.

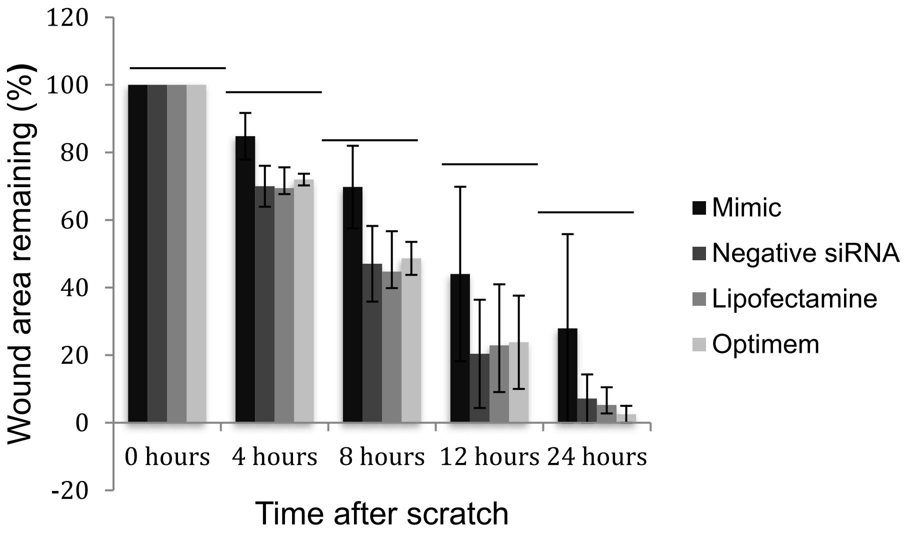

Figure S5. Percentage wound area remaining during scratch wound assay using a miR-429 mimic. The average percentage wound remaining for each condition is recorded at 0, 4, 8, 12 and 24 h. The data represents the mean from three separate experiments. An ANOVA test was used to look for significance between groups. A Tukey's test demonstrated that there was no significant difference between the mimic and negative siRNA groups suggesting that miR-429 does not affect wound adhesion.

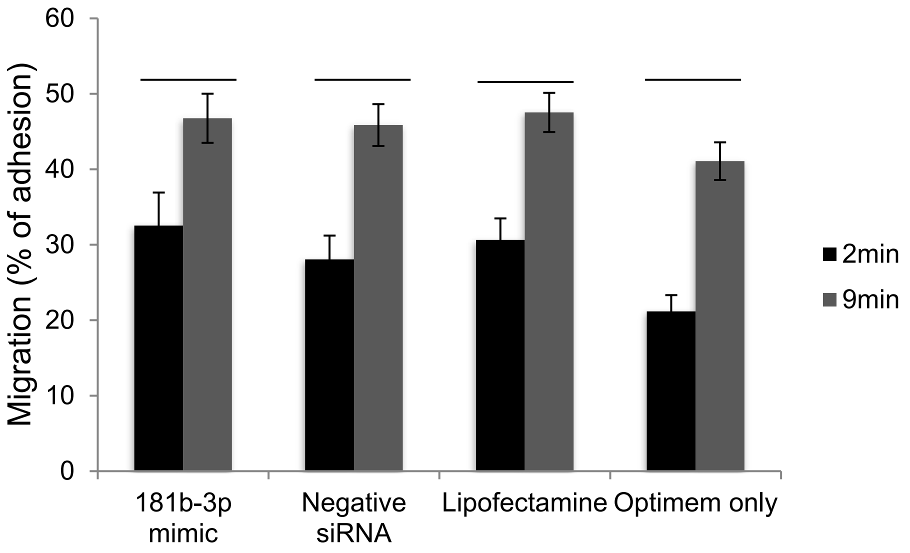

Figure S6. Static transendothelial study investigating miR-181b-3p. This chart shows the proportion of cells that have migrated during the assay at 2 and 9 min. The data represents the mean from three separate experiments. An ANOVA test was used to look for significance between groups. A Tukey's test demonstrated that there was no significant difference between the mimic and negative siRNA groups suggesting that miR-181b-3p does not affect neutrophil transmigration across pulmonary endothelium.

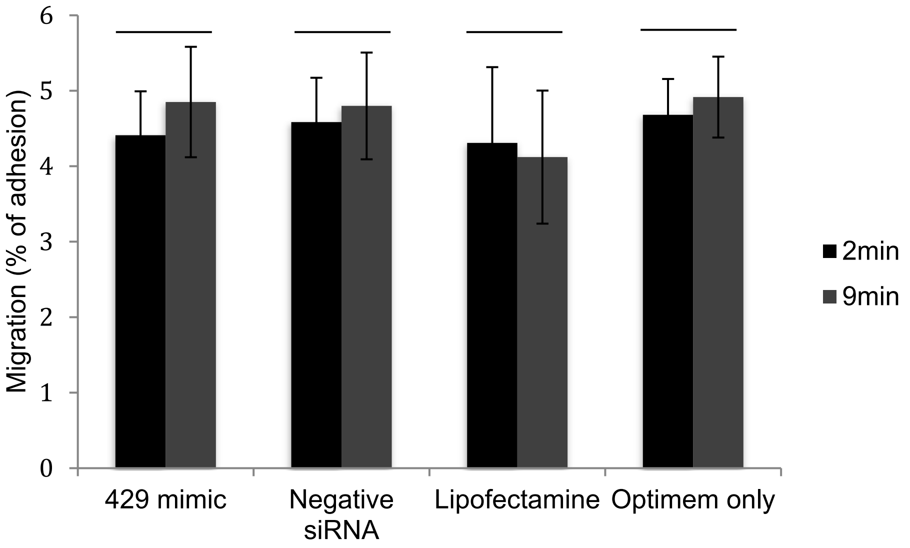

Figure S7: Static transendothelial study investigating miR-429. This chart shows the proportion of cells that have migrated during the assay at 2 and 9 min. The data represents the mean from three separate experiments. An ANOVA test was used to look for significance between groups. A Tukey's test demonstrated that there was no significant difference between the mimic and negative siRNA groups suggesting that miR-429 does not affect neutrophil transmigration across pulmonary endothelium.
